# Bibliographical

**Published:** 1882-09

**Authors:** 


### BIBLIOGRAPHICAL.

*Diseases of the Rectum: Their Diagnosis and Treatment, By William Allingham, M. D. Published by P. Blackiston, Son & Co., Philadelphia. Cloth. Pages*, 168.

A new edition of an excellent and popular work on Fistula, Hemorrhoids, Painful Ulcer, Stricture, Prolapsus, etc. From the many editions that have been sold, the author may consider himself not only an authority, but highly complimented. It has been thoroughly revised, many obscurities have been removed, and an index has been added which facilitates reference. Technical phrases have been avoided to a great extent. The first chapter is introductory, giving statistics, and classifying over 4,000 cases that the author treated, while at St. Mark's Hospital, in London. Of these 1,208 were fistula; 45 abscess; 860 internal hemorrhoids; 102 external hemorrhoids; 446 fissure; 348 syphilitic diseases of the *anus* and *rectum*; 190 ulceration, neither malignant nor syphilitic; 185 constipation; 180 puritus ani; 178 stricture of the rectum; 105 cancer of the rectum; 53 procedentia; 16 polypus without stricture; 15 hemorrhage; 14 impaction of *facèes*; 12 neuralgia; 12 dysentery; 8 spasmodic contraction of the sphincter; 7 proetitis; 5 foreign bodies in the rectum; 4 necrosis of bone; 2 rodent ulcer, and 2 vicarious menstruation from the rectum. We give the statistics to show the percentage of the different classes treated. A lucid dissertation is given in the second chapter, laying out different methods for the examination of patients. The other chapters relate to different diseases, together with the treatment and description of peculiarly treated cases. As a whole, the book is a valuable addition to the medical library.

*Nitro-Glycerine in Angina Pectoris. By William Murrell, M. D., M. R. C. P. Published by Geo. S. Davis, Detroit, Mich. Cloth. Pages*, 78

The object of this work is to give directions for the administration of nitro-glycerine as a remedy for *angina pectoris*, the principle points being illustrated by reference to cases that have been under the author's care. This work will prove valuable to practitioners, not so much for its clear analysis, but for its description of the drug and the effects it produces on the patient to whom it is administered. A history of nitro-glycerine is given, stating the date of discovery as 1847, and the name of the discoverer as Sobrero. The work contains charts showing the state of the pulse before and after the inhalation, there being ten in number, and the time is generally four minutes between each examination. The author is a firm believer in the healing properties of the drug, and from the number of cases he sites, we presume the curative powers are numerous.

HANDBOOK OF OPTHALMIC PRACTICE, *by Charles Higgins, M. D., F. R. C. S., assistant surgeon and lecturer in Opthalmic Department, Guy's Hospital. Second edition, Philadelphia: P. Blackiston, Son & Co.*, 1882. *Pages*, 116. *Price in cloth*, 50 *cents.*

This is a handsome little book, written in concise style, and admirably adapted to the use of the general practitioner.

THE OCTOBER CENTURY.—Unusually varied and interesting are the contents of the October *Century*, which closes the first year of the magazine under the new name. Articles of practical or timely value are E. V. Smalley's third paper on the New Northwest, which describes the life and natural features of the country between the Rockies and the Cascade Ranges, including the new wheat-fields of Idaho and Washington; an admirable statement of "The Growth of the United States," by Francis A. Walker, the superintendent of the last census; and an account by Judge Farman, late Consul-General at Cairo, of his "Negotiations for the Obelisk," with much that bears on the troubles in Egypt, of which the present rebellion is the grand sequel. It contains much other excellent matter, and is beautifully illustrated.

THE POPULAR SCIENCE MONTHLY FOR OCTOBER.—The October number of *The Popular Science Monthly* is one of great excellence. While all its articles deserve to be well spoken of, several of them are unusually fitted to attract attention. "Topics of the Time" discusses the rising generation at the South, burial reform, and other important questions, and the editorial departments offer a great variety of interesting criticism and information.

